# MTF-1-Mediated Repression of the Zinc Transporter *Zip10* Is Alleviated by Zinc Restriction

**DOI:** 10.1371/journal.pone.0021526

**Published:** 2011-06-27

**Authors:** Louis A. Lichten, Moon-Suhn Ryu, Liang Guo, Jennifer Embury, Robert J. Cousins

**Affiliations:** 1 Center for Nutritional Sciences, Food Science and Human Nutrition Department, College of Agricultural and Life Sciences, University of Florida, Gainesville, Florida, United States of America; 2 Department of Biochemistry and Molecular Biology, College of Medicine, University of Florida, Gainesville, Florida, United States of America; Texas A&M University, United States of America

## Abstract

The regulation of cellular zinc uptake is a key process in the overall mechanism governing mammalian zinc homeostasis and how zinc participates in cellular functions. We analyzed the zinc transporters of the Zip family in both the brain and liver of zinc-deficient animals and found a large, significant increase in Zip10 expression. Additionally, Zip10 expression decreased in response to zinc repletion. Moreover, isolated mouse hepatocytes, AML12 hepatocytes, and Neuro 2A cells also respond differentially to zinc availability in vitro. Measurement of Zip10 hnRNA and actinomycin D inhibition studies indicate that *Zip10* was transcriptionally regulated by zinc deficiency. Through luciferase promoter constructs and ChIP analysis, binding of MTF-1 to a metal response element located 17 bp downstream of the transcription start site was shown to be necessary for zinc-induced repression of *Zip10*. Furthermore, zinc-activated MTF-1 causes down-regulation of *Zip10* transcription by physically blocking Pol II movement through the gene. Lastly, ZIP10 is localized to the plasma membrane of hepatocytes and neuro 2A cells. Collectively, these results reveal a novel repressive role for MTF-1 in the regulation of the *Zip10* zinc transporter expression by pausing Pol II transcription. ZIP10 may have roles in control of zinc homeostasis in specific sites particularly those of the brain and liver. Within that context ZIP10 may act as an important survival mechanism during periods of zinc inadequacy.

## Introduction

Zinc is an essential dietary component. Its functions in biology are numerous, but can be separated into three main categories: catalytic, regulatory, and structural roles. For example, greater than ten percent of the human genome codes for zinc-containing proteins [1]. Not surprisingly, deficiency of this micronutrient is associated with diverse pathology, including impaired immunity, retarded growth, neurological disorders, and delayed wound healing [2]. However, the mechanisms leading to these clinical manifestations of zinc deficiency remain elusive.

Tight-control of cellular zinc homeostasis is maintained by proteins that can affect the amount of available zinc. Metal transporters of the ZRT/ IRT-like protein (ZIP) family and zinc transporter (ZnT) family, as well as zinc-binding by metallothioneins (MTs), maintain control of intracellular zinc levels [3], [4], . Currently, 10 ZnT and 14 ZIP transporters have been identified. The ZnT proteins function in cellular zinc efflux or vesicular storage. ZnT1 was the first zinc transporter to be characterized, and is expressed in all tissues, localizing to the plasma membrane of cells [5]. Subsequent studies revealed zinc-regulated expression of ZnT1 in the intestine [6], and that zinc regulates expression of ZnT1 through activation of the metal response element-binding transcription factor MTF-1 [7].

Metal-inducible genes regulated by MTF-1 include the Metallothioneins (*Mt*), glutamate–cysteine ligase heavy chain (*cGCShc*) which codes for an oxidative stress-related protein [8], and the aforementioned *ZnT1*. MTF-1 responds to changes in intracellular zinc and other heavy-metals (e.g., Cadmium and Copper) where upon metal-occupancy there is translocation from the cytosol to the nucleus. Subsequently this transcription factor binds to metal response elements (MREs) located in the proximal promoters of metal responsive genes, resulting in an increased rate of transcription [9]–[11]. The importance of MTF-1 to zinc homeostasis and mammalian physiology is accentuated by the fact that ablation of the gene leads to severe liver degeneration and embryonic lethality by day 14 of gestation in mice [12]. Subsequent investigation of a liver-specific conditional knockout of the *MTF-1* gene in adult mice revealed a second MTF-1 regulated zinc transporter gene *Zip10* [13].

Among the 24 zinc transporters, a number have exhibited responsiveness to dietary zinc intake [3]. Of those, ZnT1 and Zip4 have received the most attention. Mechanistically, during dietary zinc restriction, down-regulation of *ZnT1* occurs through decreased MTF-1 activation and *ZnT1* promoter binding [7]. The functional outcome of less ZnT1 expression is decreased cellular zinc efflux. Concomitant to decreased ZnT1 expression is an increase in the intestinal, apically-localized zinc transporter, ZIP4, which results in increased dietary zinc absorption in response to zinc restriction [14], [15]. *Zip4* expression does not appear to be MTF-1 regulated. *Zip10* belongs to the *Zip* class of zinc transporters which oppose the ZnT transporters by increasing cellular zinc concentrations through plasma membrane zinc uptake or vesicular efflux [3], [4], [16]. Activation of MTF-1 by cadmium, and subsequent binding to the *Zip10* promoter, led to inhibition of *Zip10* expression [13], an observation opposite to that seen with *ZnT1*. Similar observations have been made for *Zip10* expression using different model systems [17]–[20]. In silico promoter examination and EMSA analysis revealed one functional MRE located at +17 relative to the *Zip10* transcription start site [13]. This was the first demonstration of metal-dependant transcription repression of a gene by MTF-1. Here we demonstrate the dynamic responsiveness of *Zip10* in mouse liver and brain to zinc restriction and excess. Furthermore, ChIP analysis revealed in vivo association of MTF-1 with the *Zip10* promoter in mouse hepatocytes after zinc supplementation. In addition, *MTF-1* siRNA and luciferase reporter constructs show repression of *Zip10* expression occurs via MTF-1 activity and DNA binding to the downstream MRE of *Zip10*. These experiments identify ZIP10 as an important zinc transporter involved in maintenance of brain and liver zinc homeostasis, as well as revealing a novel role for MTF-1 in the regulation of zinc transport.

## Materials and Methods

### Mice and Treatments

Adult male CD-1 strain mice (Charles River Breeding Laboratories) that ranged in age from 6 to 8 weeks were housed and fed as described in detail [15]. Mice were fed either a zinc deficient (ZnD: <1 mg/kg) or zinc adequate (ZnA: 30 mg/kg) diet, individually for up to 21 days. Additionally, ZnD mice were fed a supplemental (ZnR: 180 mg/kg) zinc diet for zinc repletion. Blood was collected by cardiac puncture under anaesthesia for measurement of the serum zinc concentration by atomic absorption spectroscopy. The protocol was approved by the University of Florida Institutional Animal Care and Use Committee (protocol #200903621).

### Antibody Production

The peptides KRNHKCDPEKE and SASLSLPLVLQSG were used to produce antibodies in rabbits to ZIP10 and MTF-1, respectively. Peptides were analyzed by mass spectrometry to assess purity. An N-terminal cysteine was added to each peptide to facilitate conjugation to keyhole limpet hemocyanin (KLH) and for conjugation to Sulfo-link (Pierce) matrix for affinity chromatography. These methods are described in [6], [15], [18]. Specificity of the ZIP10 antibody has been demonstrated previously [18]. MTF-1 antibody was ChIP-grade and was purchased from Santa Cruz Biotechnology (SC-26844).

### Immunocytochemistry of Mouse Liver and Hepatocytes

Liver sections from mice that had been anesthetized and exsanguinated were fixed with 10% formalin in PBS, embedded in paraffin, cut as 5-µm sections, and mounted. Incubation with the affinity purified primary antibodies (10 µg/ml) was followed by addition of anti-rabbit IgG-Alexa 594 conjugate or anti-goat IgG-Alexa 488 conjugate (Molecular Probes). Detection and visualization of ZIP10 in primary hepatocytes and AML12 hepatocytes was performed as previously described [21]. As negative controls, respective peptides were incubated with the primary antibody before exposure to the tissue section, as previously described [15], [18]. Counterstaining of nuclei was performed with 4′,6-diamidino-2-phenylindole (DAPI).

### Immunohistology of Mouse Brain

The mice were anesthetized and then perfused intracardially with 4% paraformaldehyde. After perfusion, the brain was fixed for 24 h in 4% paraformaldehyde and transferred to 70% ethanol until being embedded in paraffin. The entire brain was serially sectioned (4 µm sections), and every 10th section was stained with hematoxylin and eosin (H&E). Immunostaining of adjacent sections was performed according to a standard streptavidin-peroxidase procedure and as previously described [22]. Endogenous peroxidise activity was suppressed with 3% H_2_O_2_. Tissues were incubated with the affinity purified ZIP10 primary antibody (1∶1000 dilution) in 3% normal serum and 0.1% Triton-X, followed by a rabbit anti-goat secondary biotinylated antibody (Vector, Burlingame, CA) and lastly were incubated with avidin-biotin-peroxidase, and visualized with the DAB chromagen (Vector, Burlingame, CA).

### Western Blot Analysis of Membrane Proteins

Liver samples were homogenized immediately in cold buffer (20 mM Hepes, pH 7.4/1 mM EDTA/300 mM mannitol) containing a protease inhibitor mixture (Pierce) by using a Potter-Elvehjem homogenizer. Membrane protein preparations were prepared by centrifugation (100,000× g) after nuclei and debris were first removed by centrifugation at 1,000× g. The proteins were resolved by PAGE using 10% gels and transferred to a nitrocellulose membrane (Schleicher and Schuell), then incubated with the appropriate affinity-purified antibody, and finally visualized by chemiluminescence detection. These methods have been described previously [6], [15]. Antibodies against actin (Sigma) and Na/K ATPase (Santa Cruz) were used to analyze protein loading of total cell lysate or the membrane fraction, respectively.

### Hepatocyte isolation and culture

Mice were anesthetized and hepatocytes isolated as previously described [21]. Viability was assessed with Trypan Blue. Only hepatocytes suspensions with >95% viability, were used in experiments. Following selective attachment of parenchymal cells, medium in each well was changed, and these culture conditions were continued for 18–22 h.

### Cell Culture and treatments

AML12 mouse hepatocytes (ATCC) were grown in DMEM/F-12 containing 10% (v/v) FBS, dexamethasone (40 ng/mL), and ITS (insulin, Tf, selenium) supplement (BD Biosciences). The Neuro 2A cells (ATCC) were maintained in DMEM with 10% FBS. Medium also contained penicillin, streptomycin, and amphotericin B (Sigma). The hepatocytes and neuroblastoma cells were maintained as previously described [21]. In some experiments the cultures contained either TPEN (4 µM), a membrane permeable chelator or DTPA (50 µM), a hydrophobic membrane impermeable zinc chelator [23], [24].

### Relative mRNA quantification

Total RNA was isolated from liver, brain, AML12 hepatocytes, and Neuro 2A cells using TRIzol (Invitrogen) and treated with Turbo DNase (Ambion) to eliminate any DNA contamination. Quantitative PCR was used to determine the relative amount of *Zip10* mRNA. DNA amplification was ruled out through no RT reactions. The PCR reaction conditions were 95°C for 10 min, followed by 40 cycles of 95°C for 15 s, 60°C for 60 s, and one final cycle at 60°C for 60 s. After PCR, melting curves were acquired to ensure that a single product was amplified during the reaction. The primers used for PCR amplified the exon 10 region of *Zip10* mRNA and were as follows: sense primer, 5′- TGGCTTACATAGGAATGCTCATAGG-3′, and antisense primer, 5′- TGCGAAGATCCAGAGTGTGATG-3′, Zip10 hnRNA sense primer 5′–TGTTGCAGTCAAAAATCTGAAG–3′, and antisense primer 5′–CCATGTGAATGACCATGTCC–3′. Transcript abundances were normalized to 18s rRNA sense primer, 5′-AGTCCCTGCCCTTTGTACACA-3′, and antisense primer, 5′-GATCCGAGGGCCTCACTAAAC-3′, or TATA-binding protein (TBP) (ABI). The results are expressed as arbitrary units of *Zip10* normalized to 18S rRNA, or TBP mRNA, where stated and based on a RNA standard curve. The PCR reactions were performed in duplicate for each sample, and samples were collected from at least three independent experiments.

### ChIP Analysis

Chromatin immunoprecipitation was performed as described previously [21], [25]. The reaction mixtures were incubated at 95°C for 10 min, followed by 40 cycles of amplification at 95°C for 15 s and 60°C for 60 s. Primers for *Zip10* were Zip10TSS sense primer 5′-AGAATACACGACTGGGTGCAG-3′, and antisense primer 5′–TGGGGGAGGGAATGTAAAC–3′; Zip10 exon2 sense primer 5′–TGCTCCAAGTGACACAAAGC–3′, and antisense primer 5′–CGCTAGTTACCGCACTTAACG–3′; Zip10 exon10 sense primer 5′–AATGGAGCGGTCAGTGTTTG–3′, and antisense primer 5′–GTGGCATGGGATGTAAACAG–3′; Zip10 Upstream sense primer 5′–AATAAGGCCCAGCACTGAAG–3′, and antisense primer 5′–ACATTTACCCCTGTGGCATC–3′.

### Zip10 promoter construction and mutagenesis

The Zip10 promoter construct was cloned (−2 kb to +1 kb relative to the Zip10 TSS) from BAC DNA (RP23-374B12), using PCR with primers containing EcoRV and HindIII sites appended to the 5′ ends. The PCR product was then subcloned into the pGemT–Easy plasmid using T/A cloning and further inserted into the pGL4.11 plasmid (Promega) by using the same EcoRV and HindIII restriction sites, immediately upstream of the luciferase gene. Plasmid constructs were confirmed by DNA sequencing. The Zip10 Luc mutant construct containing the 3 kb promoter was created using site-directed mutagenesis (Quick Change II, Startagene). The oligonucleotides used to generate the mutation of the MRE site were 5′–GTACCGAGCGGAGAGGAGAGGCCTACGGCACTCG–3′ and 5′–CGAGTGCCGTAGGCCTCTCCTCTCCGCTCGGTAC–3′. Mutation of the MRE site introduced a novel StuI restriction site that was used for screening of mutated clones. Further confirmation of mutations and integrity of promoter fragments was performed by DNA sequencing. MRE sequences were identified via the Genomatix and USCS Genome Bioinformatic databases.

### Transfection and luciferase assay

AML12 mouse hepatocytes (American Type Culture Collection) were seeded at 1×10^5^ cells/well in 24-well plates culture conditions were described previously [21]. Transfection began 12 h after seeding with 15 nM (final concentration) of siRNA for mMTF-1 or mZip10 (Smart Pool, Dharmacon) using HiPerFect transfection reagent (Qiagen), and was carried out for 48 h. For the luciferase assays, AML-12 cells were seeded in 12- or 24-well plates and transfected with pGL4.11 plasmid and the *Renilla* luciferase control plamids pRL-SV40 or pGL4.73 (Promega) by using Effectene reagent (Qiagen). After 48 h of incubation, the medium was replaced by medium with or without 40 µM ZnSO_4_ or 4 µM TPEN for 4 h. Luciferase activities were measured with a Dual-Luciferase Assay System (Promega) with a SpectraMax M5 (Molecular Devices) in luminometer mode following the manufacturer's protocol. The raw values of firefly luciferase were normalized to *Renilla* luciferase that had been transfected concurrently in all the assays to correct for differences in transfection efficiency. The Renilla luciferase was stable throughout the experiments, and not significantly affected by zinc status. The promoter activity assays were measured in triplicate in each experiment and shown as fold-change relative to control. At least three sets of independent experiments were performed for each set of constructs.

### Zinc uptake by hepatocytes

AML12 hepatocytes were incubated as above with 5 µM FluoZin-3AM (Invitrogen), a cell-permeable zinc fluorophore, in serum-free medium for 30 min. Intracellular zinc accumulation was measured as previously described [26].

### Statistical analysis

Data are presented as the means ± S.D. Significant changes between two groups were determined by Student's t-test. Two-way ANOVA and Bonferroni's post-test were used for multiple comparisons. Statistical significance was set at p<0.05.

## Results

### Dietary zinc modulates Zip10 expression in mice

**Figure 1:**
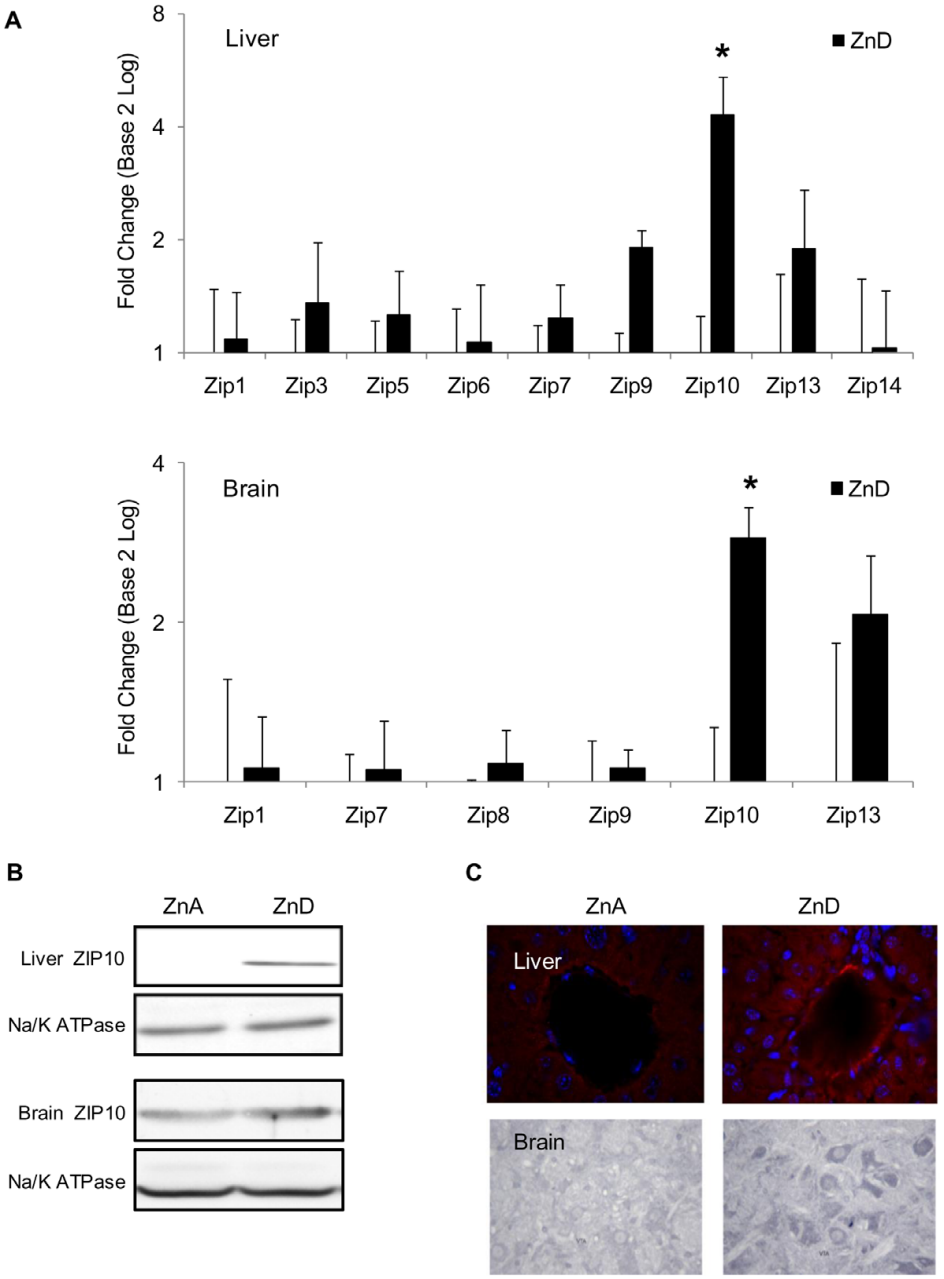
Restriction of dietary zinc increases Zip10 expression in liver and brain. (**A**) Total RNA extracts from liver and brain tissue of ZnD or ZnA mice were analyzed by qRT-PCR. Values shown are for those Zip mRNAs up-regulated by dietary zinc restriction. They are expressed as relative to 18 rRNA in a log_2_-scale (mean ± SD, n = 4). Statistical significance at P<0.05 or greater is indicated with an asterisk since values from the ZnA mice are expressed as 1.0 only the SD is shown. (**B**) Plasma membrane protein fractions from liver (**upper panel**) and brain (**lower panel**) tissues of ZnD or ZnA mice were isolated and separated by SDS-PAGE. Western blotting was performed by using an anti-ZIP10 antibody. Blots were then stripped and re-probed using an anti-Na/ K ATPase antibody. Representative blots are shown. (**C**) Sections of liver (**upper panel**) and brain (**lower panel**) from ZnA and ZnD mice. Immunolocalization of ZIP10 was performed by using the anti-Zip10 antibody, and visualization was achieved with an Alexa fluor594 secondary antibody or Horseradish immunoperoxidase. Representative regions of the sublentic amygdala (SLEA) and lateral globus pallidus (LGP) and the mesencephalic ventral tegmental area (VTA) of ZnA and ZnD mice were examined.

By utilizing the gene expression portal BioGPS, we narrowed our search for tissues expressing *Zip10* at high levels to the liver (median value) and the brain (10-fold median value) [27]. For comparative purposes, our initial study of *Zip10* began by screening changes in mRNA expression of all Zip transporters in liver and brain tissue of mice following three weeks of dietary zinc deficiency. This murine model of zinc restriction has been used extensively [15], [18]. Evidence for creation of zinc deficiency is the reduction in serum zinc concentration. Although changes in other transporters were observed (e.g., *Zip9* and *Zip13*), the most robust change in gene expression was seen in *Zip10* (Figure 1A). Average C_t_ values for the Zip transporter mRNA assays are provided in Table S1. Analysis of relative transcript abundance by qPCR revealed a significant 2-fold increase in liver, and 3-fold increase in brain *Zip10* expression from mice maintained on the zinc-deficient diet. Of interest is the relative 30-fold higher Zip10 mRNA expression in brain versus liver tissue (Figure S1). Western analysis of ZIP10 protein abundance from liver and brain (Figure 1B) showed similar increases in expression from mice fed the zinc-deficient diet. These increases were 1.6 fold or greater. In addition, immunohistochemical analysis with the anti-ZIP10 antibody revealed a mainly cytoplasmic distribution of ZIP10 in liver of ZnA mice (Figure 1C). The cytoplasmic distribution of ZIP10 is interpreted as ZIP10 associated with endocytotic processing and/or movement of the protein to the plasma membrane. However, under ZnD conditions, ZIP10 is localized to distinct intracellular membranes and the plasma membrane. The most prominent areas of ZIP10 localization in the forebrain were the posterior lateral, posterior internal and the posterior medial striatum terminalis. The lateral globus pallidus (LGP), sublentic amygdala (SLEA) and dorsal lateral septal nuclei also showed marked ZIP10 expression. Staining was markedly increased in those sections from brains of the ZnD mice. The ventral tegmental area (VTA) was also highly immunopositive for ZIP10 and was increased in the ZnD mice. These results indicate that Zip10 is abundantly and differentially expressed in both liver and brain tissue of mice and responds to dietary zinc status.

### Zip10 responds quickly to dietary zinc repletion

**Figure 2:**
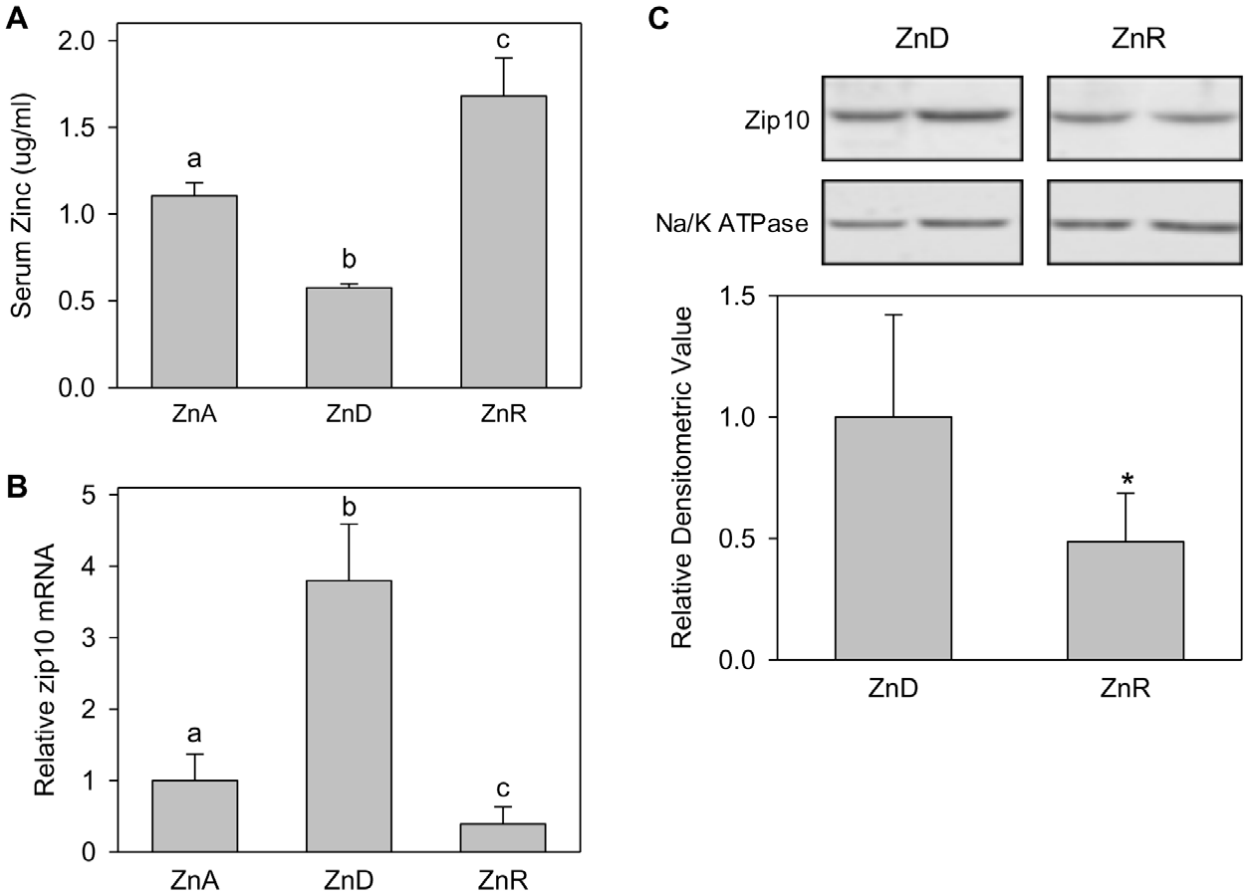
Dietary zinc repletion decreases Zip10 expression. (**A**) Serum zinc concentrations were measured in mice fed the ZnD or ZnA diets for 7 d, followed by one group of ZnD mice receiving a zinc-repletion diet (ZnR) for 1 d. (**B**) Relative abundance of Zip10 mRNA between ZnA, ZnD, and ZnR groups is shown normalized to 18s rRNA. Values are given as means ± SD. Values with a different superscript are significantly different at *P*<0.05 or greater, *n* = 5. (**C**) Representative western blot analysis (**upper panel**) and relative densitometry normalized to Na/K ATPase (**lower panel**) of total liver membrane proteins showing the decrease in ZIP10 abundance upon zinc repletion.

After 7 d of zinc depletion, the serum zinc concentration dropped to approximately one-half normal levels (Figure 2A). The up-regulation of Zip10 mRNA was shown with this shorter zinc restriction (Figure 2B). By providing animals with supplemental zinc (ZnR) in the diet for 1 d following the 7-day depletion (ZnD), Zip10 mRNA abundance was significantly decreased, while serum zinc was significantly increased. The changes in serum zinc concentrations serve as a positive control of the in vivo effectiveness of the nutritional treatments used. Western blot analyses of liver membrane proteins showed that, by day 7 of depletion, ZIP10 was markedly increased (Figure 2C). However, following only 1 d of repletion of the mice with the ZnR diet, ZIP10 levels decreased dramatically. These results show that the *Zip10* gene is capable of rapidly responding to the dietary zinc supply.

### Regulation of Zip10 expression by zinc

**Figure 3:**
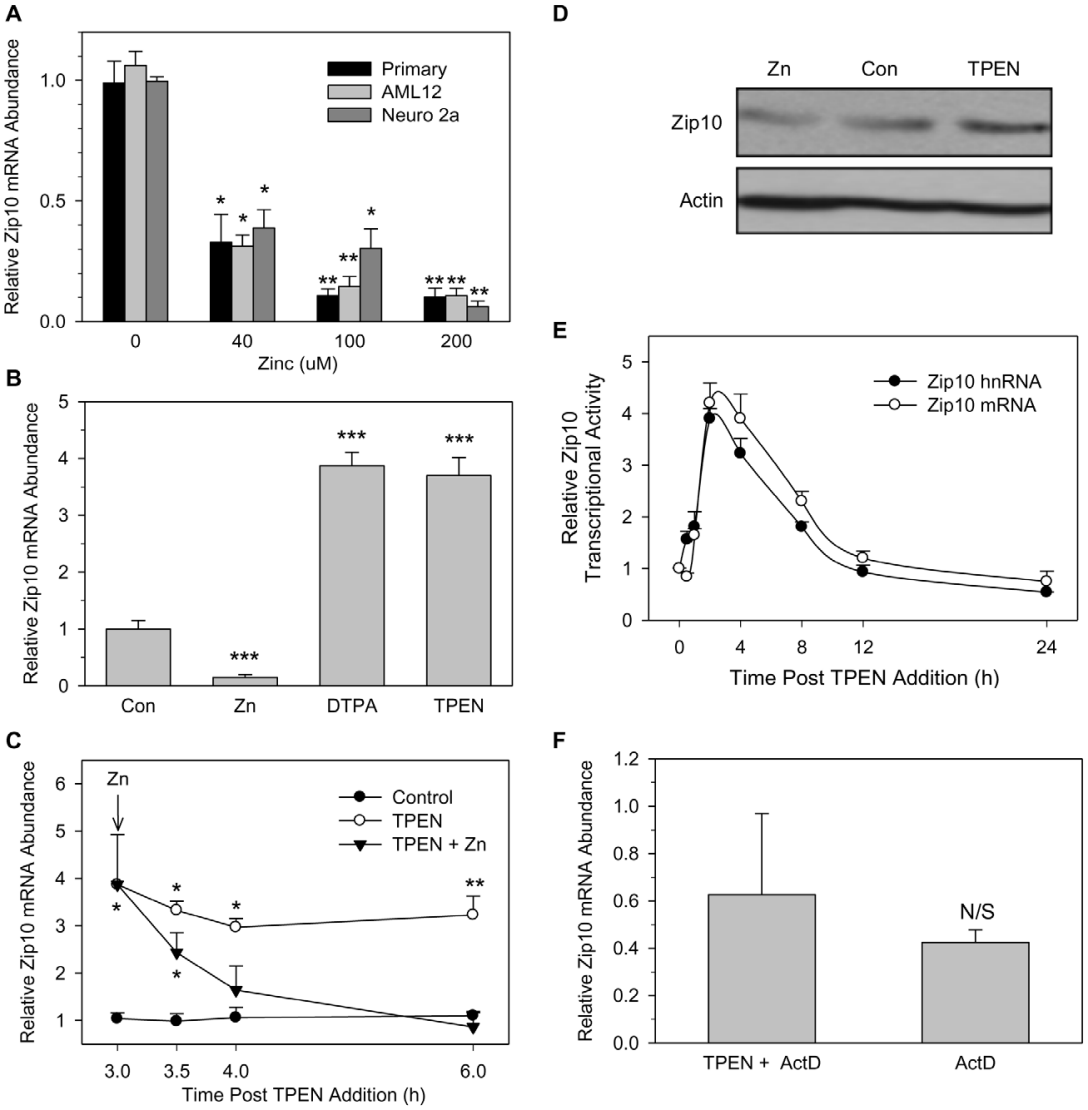
Zinc availability modulates Zip10 expression. (**A**) The relative abundance of Zip10 mRNA was measured by qRT-PCR and compared in primary and AML12 mouse hepatocytes, and Neuro2A cells after being supplemented with increasing amounts of zinc (0–200 µM) for 3 h. (**B**) AML12 hepatocytes were treated for 3 h with 40 µM zinc, or one of the zinc chelators DTPA (50 µM) or TPEN (4 µM) to deplete cellular zinc levels. (**C**) AML12 hepatocytes were pre-treated with TPEN (4 µM) for 3 h to 6 h to increase Zip10 mRNA abundance. Then a subset of hepatocytes was given supplemental zinc (40 µM) for 3 h. Cells were harvested at the times indicated and Zip10 mRNA levels were measured by qRT-PCR. (**D**) Western analysis of AML12 hepatocytes after treatment with 40 µM zinc or 4 µM TPEN. Representative blots are shown. (**E**) Time-course comparing Zip10 mRNA and hnRNA relative to TBP mRNA in AML12 hepatocytes post-TPEN treatment. (**F**) Zip10 mRNA abundance relative to TBP mRNA after pre-treatment of hepatocytes with Actinomycin D for 1 h followed by exposure to TPEN for 3 h. Data are expressed as mean ± SD, and values with a different superscript are significantly different at *P*<0.05 (*n* = 3). Data are from a representative experiment that was repeated multiple times.

To further investigate the relationship between *Zip10* and zinc, we used primary and AML12 mouse hepatocytes as models of liver cells, and Neuro-2A cells as representative neuronal cells. Treatment of these cell types with increasing amounts of zinc resulted in a dose-dependant repression of *Zip10* transcript abundance (Figure 3A). Because no significant differences were seen between the responses of the cell lines tested, the AML12 cell line was used for subsequent experiments. To simulate zinc deficiency, AML12 hepatocytes were incubated with either TPEN (4 µM) or DTPA (50 µM) both zinc chelators, which lead to a 4-fold increase in *Zip10* mRNA levels (Figure 3B). Furthermore, the response of the hepatocytes to zinc repletion was similar to the in vivo liver response (Figure 3C). The AML12 hepatocytes responded very quickly to medium supplemented with zinc by down-regulating Zip10 expression within 30 min following zinc addition.

The differential response of the hepatocytes to zinc availability was also examined at the protein level. To determine ZIP10 protein expression, 8 h following zinc or TPEN treatment, membrane proteins were isolated from the AML12 hepatocytes for western analysis. ZIP10 abundance (Figure 3D) closely followed that found for *Zip10* mRNA. A comparable increase in ZIP10 was observed with DTPA (data not shown).

As an initial approach to determine whether zinc is modulating Zip10 expression through a transcriptional mode of regulation, we measured the abundance of Zip10 mRNA as well as short-lived hnRNA [28] post TPEN treatment (Figure 3E). In agreement with zinc deficiency inducing Zip10 expression by increasing gene transcription, Zip10 hnRNA mirrored the mRNA. Furthermore, AML12 hepatocytes were exposed to 5 µM TPEN for 3 h in the presence or absence of 5 µg/mL actinomycin D, an inhibitor of RNA synthesis. As shown by the results of qPCR (Figure 3F), cotreatment with actinomycin D blocked the TPEN-induced increase in Zip10 mRNA.

### Zinc regulated expression of Zip10 occurs through activation of MTF1

**Figure 4:**
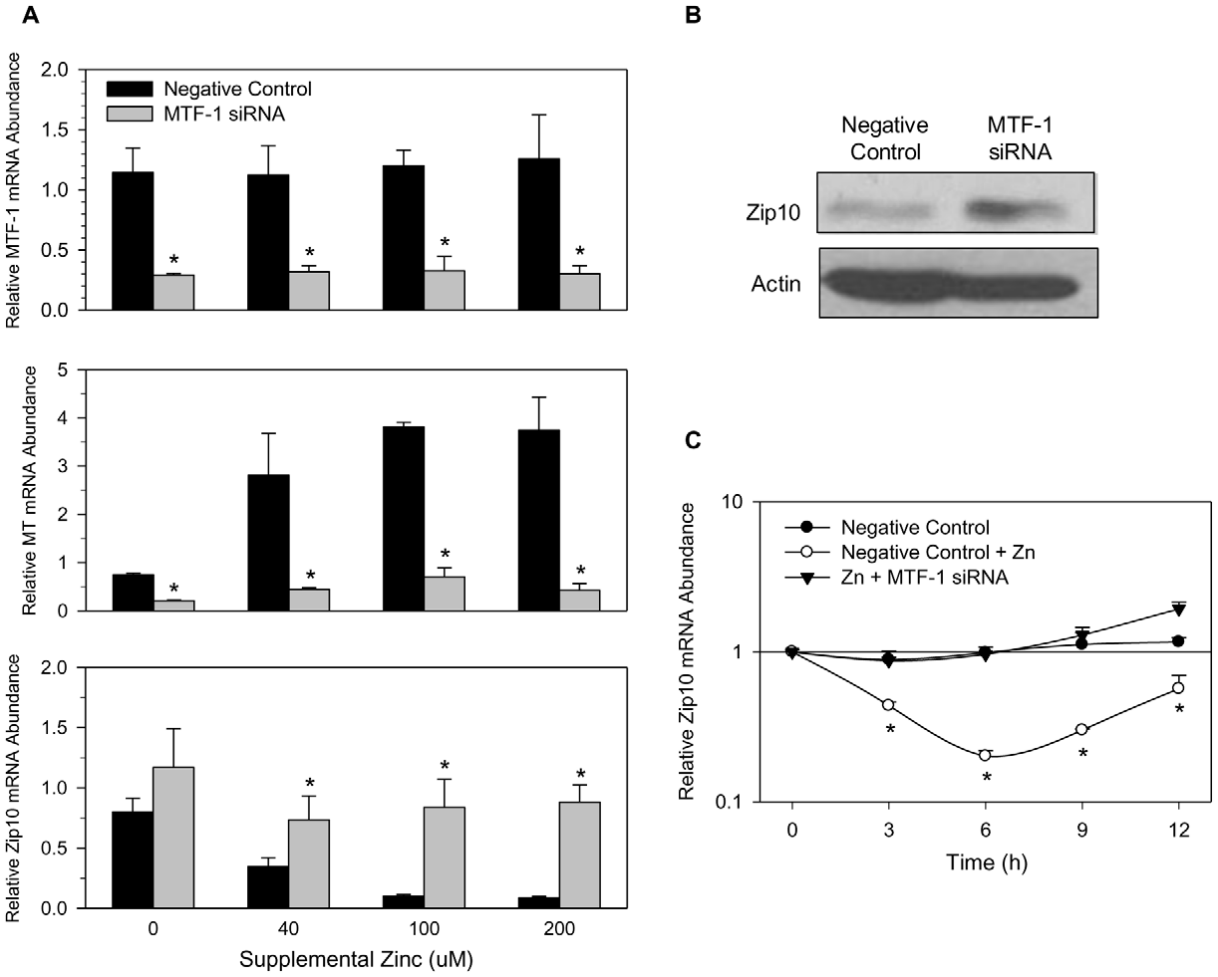
MTF-1 knockdown increases Zip10 expression. (**A**) AML12 hepatocytes were transfected with MTF-1 siRNA and allowed to incubate for 48 h. Increasing amounts of zinc were subsequently added to the culture medium, and hepatocytes were incubated for an additional 3 h. The relative abundance of MTF-1 (**upper panel**), MT (**middle panel**), and Zip10 mRNAs (**lower panel**) were analyzed by qRT-PCR. (**B**) The effect of MTF-1 knockdown on ZIP10 protein levels was further analyzed by western blotting with actin used as a loading control. (**C**) Time-course measuring the response of Zip10 to zinc addition following MTF-1 transfection, as described above. Values with a different superscript are significantly different from the corresponding negative control value at *P*<0.05 or greater. *n* = 3.

Mechanistically, exposure of cells to zinc causes a rapid translocation of MTF-1 from the cytoplasm to the nucleus [9], [10] and up-regulation of the zinc-responsive genes *Mt* and *ZnT1* occurs via binding of MTF-1 to the corresponding proximal-promoter region. However, through EMSA analysis, MTF-1 was shown to bind to a downstream MRE of *Zip10* (i.e., +17 relative to the TSS) after Cd exposure [13]. However, this downstream MRE of Zip10 was proposed through bioinformatic analysis, not direct sequencing. Therefore, to investigate the role of this putative downstream MRE in Zip10 regulation, we first performed 5′ RACE from total RNA taken from liver and brain tissues of ZnD animals. The results revealed that (Figure S2) a single band was amplified, indicating that one TSS exists. Upon sequencing, the transcript matched exactly with the public record NM_172653.2 with one MRE located 17 bp downstream of the TSS. Our next step was to determine if MTF-1 was mediating the zinc sensitivity of Zip10. By using siRNA directed against MTF-1, a clear decrease in MTF-1 expression was evident 48 h post siRNA transfection (Figure 4A). As a positive control for zinc sensitivity and MTF-1 transcriptional control, *Mt* mRNA levels were measured post MTF-1 siRNA transfection . Following transfection of MTF-1 siRNA, the ability of zinc to induce *Mt* expression was severely hindered. After finding a significant decrease in MTF-1 expression and a lack of increase in *Mt* expression, we examined the *Zip10* mRNA levels following zinc treatment in cells lacking MTF-1. In addition, ZIP10 protein levels were elevated in cells lacking MTF-1 (Figure 4B). Moreover, the knockdown of MTF-1 was able to reverse the dose-dependent repression of *Zip10* by zinc, and this reversal was maintained for 12 h (Figure 4C).

### MTF-1 binding to the Zip10 MRE is required for gene repression

**Figure 5:**
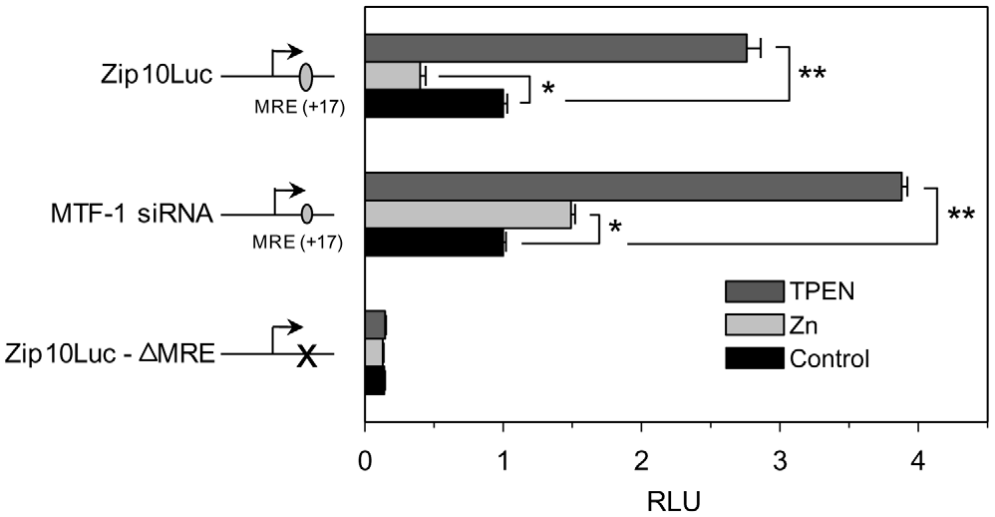
Zinc regulates Zip10 expression in an MTF-1 dependant manner. A 3 kb region of the Zip10 promoter was cloned in front of the *firefly* luciferase gene of pGL4.11 and co-transfected with *renilla* luciferase into AML12 hepatocytes. For one series of experiments MTF-1 siRNA was co-transfected with the luciferase vectors. In another series of experiments the MRE was mutated to a non-responsive StuI restriction site. Forty eight h post-transfection, cells were incubated for 3 h with 40 µM zinc or 4 µM TPEN. The data represent relative mean *firefly/ renilla* activity from a representative experiment that was replicated. Values with a different superscript are significantly different at *P*<0.05(*) or greater(**) (*n* = 3 for independent experiments).

To further investigate the roles of MTF-1 and the MRE of Zip10, we cloned a fragment comprising −2 kb to +1 kb of the Zip10 gene into the luciferase vector pGL4.11. This allowed us to determine the functional capacity of the exonic MRE located downstream of the TSS. Upon transfection into AML12 cells, the promoter activity was repressed by addition of 100 µM zinc (Figure 5A). In contrast, treatment of the transfected cells with TPEN Zip10 promoter activity was markedly increased. In addition, co-transfection with *MTF-1* siRNA reduced the response of the *Zip10* reporter to activation by 100 µM zinc *MTF-1* siRNA had no effect on the influence of TPEN. Further, the MRE upon being mutated lost responsiveness to zinc. These results show that the *Zip10* promoter as a 3 kb fragment which includes the MRE at +17, responds to zinc as observed in vivo with mouse liver or AML12 hepatocytes, indicating the MTF-1 is necessary for the zinc repression of the reporter.

### MTF-1 regulates Zip10 expression through obstruction of Pol II elongation

**Figure 6:**
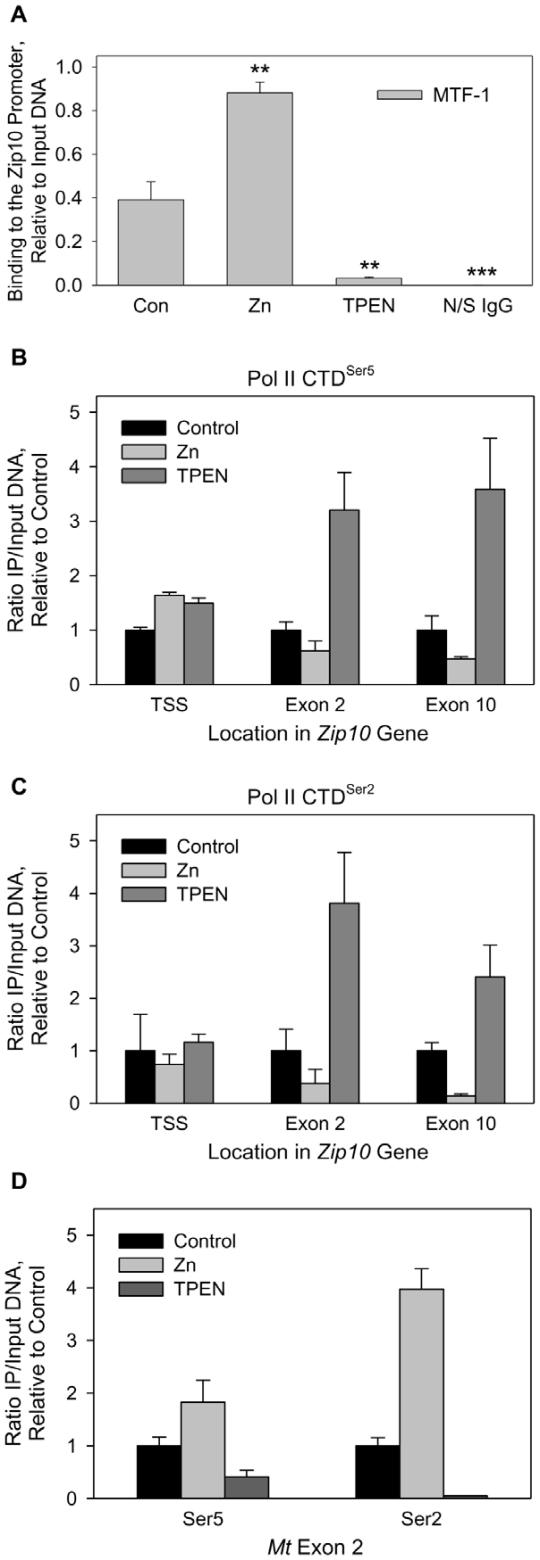
Transcriptional elongation by Pol II occurs during zinc deficiency, but not with zinc supplementation. (**A**) Association of MTF-1 with the Zip10 promoter was analyzed by using ChIP. AML12 hepatocytes were incubated with zinc or TPEN as indicated in A. The ratio of IP to input DNA was quantified by qRT-PCR. Values are significantly different at P<0.05. All data are expressed as mean ± SD. (**B**) ChIP analysis of Pol II throughout the *Zip10* gene by using an antibody specific for the Ser5 phosphorylated form of Pol II, 3 h after incubation of the AML12 hepatocytes with 40 µM zinc or 4 µM TPEN. (**C**) ChIP analysis of the elongating form of Pol II, phosphorylated at Ser2. Cells were treated as in **A**. (**D**) Analysis of Pol II association throughout the *Mt* gene. A non-specific rabbit IgG was used as a negative control for both **B** & **C**. Data for **B–D** were plotted as the ratio of immunoprecipitated DNA to a 1∶20 dilution of input DNA. A representative experiment is shown and replicated two times. Background immunoprecipitation levels were always below a ratio of 0.01 (to input DNA). Values with a different superscript are significantly different at *P*<0.05 (*n* = 3).

In an attempt to determine if MTF-1 is responsible for *in vivo* zinc-regulated expression of *Zip10*, we exposed the hepatocytes to zinc restricted and zinc supplemented conditions (i.e., 4 µM TPEN and 100 µM zinc, respectively) and analyzed the protein-DNA interactions through ChIP (Figure 6A). Binding of MTF-1 to the *Zip10* promoter was increased by almost 3-fold after 1 h of zinc treatment, and decreased to 25% after TPEN treatment. No binding was detected using a non-specific IgG in place of the MTF-1 antibody.

After establishing a relationship between MTF-1 DNA binding and Zip10 repression, we investigated the possibility that MTF-1 physically obstructs Pol II movement, preventing active transcription under conditions of adequate cellular zinc. ChIP assays were utilized to immunoprecipitate the initiating and elongating forms of Pol II. Further analysis by qPCR was conducted by amplification of the TSS, exon 2, and exon 10 of *Zip10*. The c-terminal domain (ctd) of Pol II is phosphorylated at Ser5 during initiation of transcription at the promoter of active genes [29]. Under conditions of both zinc restriction and zinc supplementation of the cells, an equal amount of Ser5-P Pol II is associated with the TSS of *Zip10* (Figure 6B), even though levels of MTF-1 at the promoter are higher under the supplemented conditions (Figure 6A). Active gene transcription involves a switch from the Ser5-P ctd to the Ser2-P ctd of Pol II for elongation. Amplification of either exon 2 or exon 10 of *Zip10*, under zinc restricted (TPEN) conditions show elongation activity of Pol II, through increased Ser2-P Pol II association with the *Zip10* gene (Figure 6C). In contrast, the supplementation with 40 µM zinc produced no evidence of Ser2-P or Ser5 Pol II association. Taken together, the results from the *Zip10* TSS and downstream exons show that Pol II is recruited to the TSS even under zinc supplemented conditions, but elongation occurs mainly under zinc-restricted conditions. Furthermore, the *Mt* gene reacts positively to zinc supplementation through activation and binding of MTF-1 to the promoter. Therefore, *Mt* serves as a positive control for transcriptional activity enhanced by MTF-1. In direct contrast to the lack of Ser2-P association with *Zip10*, under zinc supplemented conditions, but not zinc restricted conditions the Ser2-P isoform of Pol II was detected downstream of the *Mt* TSS (Figure 6D). These data suggest that MTF-1 binding to the MRE at +07 of *Zip10* creates an obstacle that prevents Pol II movement from the TSS through the downstream remainder of the gene.

### Zip10 functions as a zinc importer

**Figure 7:**
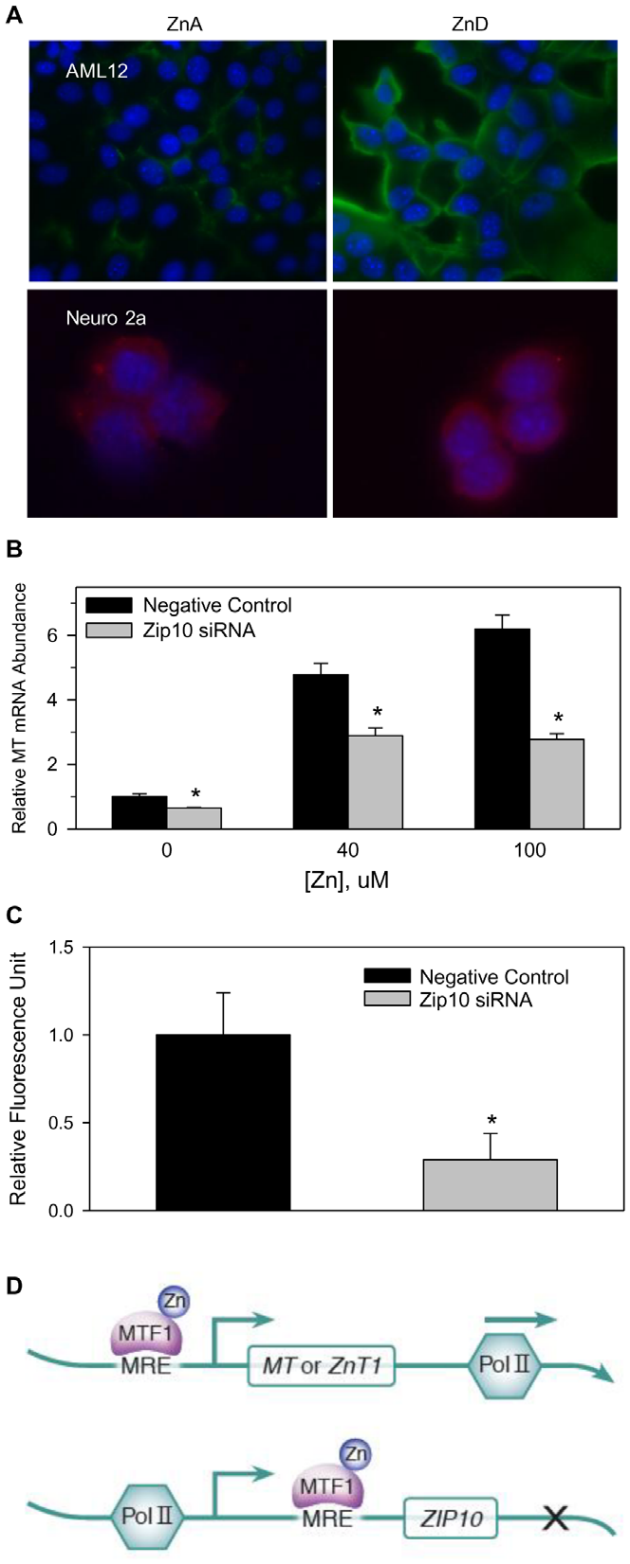
ZIP10 localizes to the plasma membrane and facilitates zinc uptake. (**A**) AML12 hepatocytes and undifferentiated Neuro 2A cells were incubated without (ZnA) or with 4 µM TPEN (ZnD). AML12 hepatocytes were incubated with the affinity purified ZIP10 antibody and detected by addition of anti-rabbit IgG-Alexa 488 conjugate. The Neuro 2A cells were incubated with the same ZIP10 antibody and anti-rabbit IgG Alexa 594 conjugate. (**B**) AML12 hepatocytes were transfected with Zip10 siRNA and 48 h later were incubated with 0, 40, or 100 µM zinc for 30 min. MT mRNA was analyzed by qRT-PCR, and normalized to 18s rRNA. (**C**) Hepatocytes were transfected with Zip10 siRNA or negative control siRNA as above. Intracellular labile zinc was detected with FluoZin-3AM fluorescence assay and reported as relative fluorescence units. Values with a different superscript are significantly different at *P*<0.05 (*n* = 3). (**D**) Model depicting the influence of the MTF-1 transcription factor based upon placement of MRE consensus sequence. Zinc occupancy causes MTF-1 translocation to the nucleus. MRE placement in the *MT or ZnT1* genes allows Pol II movement for transcription initiation and elongation. MRE placement in Zip10 allows initiation, but MTF-1 binding to the MRE prevents movement of Pol II preventing elongation. During zinc restriction MTF-1 does not move to the nucleus allowing *Zip10* transcription to proceed.

To better understand the physiologic role of ZIP10, we first identified its localization in AML12 hepatocytes. ZIP10 protein was detected at the plasma membrane of AML12 hepatocytes as well as primary hepatocytes (data not shown) under zinc-deficient (ZnD) conditions (Figure 7A). A similar increase of ZIP10 at the plasma membrane of undifferentiated Neuro 2A cells under zinc-deficient (ZnD) conditions was observed (Figure 7A). Using MTmRNA as a sentinel indicator of increased intracellular zinc, Zip10 knockdown with siRNA markedly reduced MT expression (Figure 7B). Furthermore, by using the zinc indicator FluoZin-3AM, we were able to detect an increase in zinc-associated fluorescence that was not seen when Zip10 protein expression was knocked down (Figure 7C). These data reveal a role for ZIP10 in zinc uptake across the plasma membrane of hepatocytes.

## Discussion

In the present study we demonstrate that MTF-1 is an integral part of ZIP10-related cellular zinc homeostasis in the liver and brain both during zinc restriction and zinc excess. By using intact mice and isolated murine hepatocytes we show that MTF-1 can act as a repressor of *Zip10* expression under normal physiologic levels of zinc. Zinc depletion alleviates MTF-1 associated down regulation of Zip10, allowing enhanced *Zip10* transcriptional activation.

A particularly interesting finding in our studies is the large relative difference in *Zip10* transcript abundance between the brain and the liver, although the magnitude of responsiveness to zinc deficiency is the same. Dietary zinc deprivation causes a decrease in serum zinc concentration, but results in only small reductions in the zinc context of most peripheral tissues [2]. Similarly, the concentration of zinc in the brain overall does not vary much under zinc restriction and is therefore tightly regulated [30], [31]. However, considering the neurobiology of zinc [32], region-specific localization of zinc occurs [33]. We observed significant upregulation of ZIP10 expression in the striatum, amygdala, caudate nucleus and substantia nigra as well as other regions corresponding to components of the basal ganglia. These regions play a significant role in movement control. Zinc exerts many neurobiological effects, such as modulating functions of neurotransmitter receptors in the brain [34]. Alterations in zinc homeostasis in the brain may be involved in other neurological diseases such as Alzheimer's disease, Parkinson's disease, and amytrophic lateral sclerosis [32]. Inadequate dietary zinc intake leads to changes in behavior such as reduced activity and responsiveness to stimuli [35], [36]. Zinc restriction during infancy causes impaired learning behavior [37]. Therefore, using data from the gwascentral.org database, we speculate that perturbations in ZIP10 function or single nucleotide polymorphisms that cause loss of function or restricted function may affect brain development or the pathogenesis of neurological disorders. Furthermore, the up-regulation of ZIP10 during conditions of zinc deficiency, particularly during development, may have deleterious effects on neurological processes. The potential for increased zinc influx via ZIP10 controlled import may raise issues about the known relationship between excess zinc and neural cell death [38].

For many years, MTF-1 was thought of as purely a positive promoter of zinc-induced transcription. It was originally identified as the transcription factor responsible for activation of the sentinel zinc-responsive gene, *Mt*, which helps maintain cellular zinc balance [10], [39]. Additionally, zinc-induced MTF-1 was found to up-regulate the primary zinc exporter ZnT1 (SLC30a1) [6], [7], as well as other genes involved in detoxification of heavy metals [13]. The essentiality of MTF-1-dependent gene expression became apparent when the null mutation of the gene was found to be embryonic lethal [12]. By using a liver-specific conditional knockout of MTF-1, the first indication of a repressive role for MTF-1 in heavy-metal dependant gene expression was found [13]. Specifically, the liver-specific ablation of the transcription factor allowed for increased expression of *Zip10*.

Analysis of Zip10 mRNA by 5′ RACE revealed one gene transcript and an MRE 17 bp downstream of the lone TSS. Until recently, MREs were primarily thought to lie upstream of the TSS and promote heavy-metal gene expression, as is the case for MT and ZnT1 [7], [9], [11]. In silico analysis of MRE locations revealed that while most MREs lie upstream of the TSS in mouse genes, a significant portion can be found in introns, and some in exons [40]. Another report extended these findings by using bioinformatics and ChIP to demonstrate MTF-1 binding to MREs located downstream of the translation start site of the *Selh* and *TXNRD2* genes [41]. For our experiments we used ChIP to show zinc-induced MTF-1 association with the MRE located within the first untranslated exon of the Zip10 gene. Similar results have been observed in zebrafish where zinc induces repression of Zip10 expression via binding to an intronic MRE cluster [19]. Binding of MTF-1 to this downstream MRE provides a degree of plasticity in regulation of zinc homeostasis by regulating gene transcription in the direction opposite to the usual mechanism. An analogous observation has been made for the low-affinity zinc uptake transporter gene *ZRT2* of *S. cerevisiae*. The zinc-responsive transcriptional activator Zap1 binds to an element downstream of the *ZRT2* TATA box, repressing gene expression [42].

As a mechanism to explain the repression of *Zip10* during zinc adequate conditions of hepatocytes, we hypothesized that MTF-1 acts as a transcriptional repressor by obstructing movement of Pol II from the *Zip10* TSS. Transcription is a complex multistep process involving sequence-specific activators that recruit Pol II and general transcription factors (GTFs) to the TSS for formation of the pre-initiation complex (PIC). Transcription initiation begins with TFIIH (a GTF) phosphorylating Ser5 residues in the carboxy-terminal domain of Rpb1, the large subunit of Pol II [29]. However, in some cases Pol II stalls and does not continue to transcription elongation, but instead remains poised on the TSS allowing another level of transcriptional control [43]. Developmental and heat shock genes are examples of genes regulated by promoter-proximal pausing of Pol II [44], [45]. Zinc deficiency is associated with diverse disorders, including impaired immunity, retarded growth, brain development disorders and delayed wound healing. Therefore, a zinc transporter that is responsive to zinc e.g., Zip10 could be considered critical for maintaining zinc homeostasis and would benefit from a rapid transcription response mechanism to zinc deficiency such as Pol II pausing. This is opposite to what is found for another MTF-1 regulated gene, *Mt*, upon zinc restriction. We show here that under the zinc restricted, adequate, and supplemental conditions used in these experiments, Pol II is actively recruited to the *Zip10* TSS and is poised for transcription initiation as indicated by Ser5 phosphorylation. However, for gene transcription to occur productive elongation must ensue. This entails a shift from Ser5 phosphorylation to Ser2 phosphorylation, and recruitment of various elongation factors. Only under the conditions of zinc restriction could we detect Ser2 phosphorylated Pol II. We therefore propose that when cellular zinc is adequate, MTF-1 represses *Zip10* expression by interfering with the transition from transcription initiation to elongation by impeding the movement of Pol II.

Many reports have suggested an involvement of zinc and/or zinc transporters in cancer development and progression, such as carcinomas of the liver, lymph node, and breast [46]. The levels of zinc in the serum or affected tissues of cancer patients, is decreased in various malignancies. However, in breast cancer patients the zinc levels are lower in the serum and elevated in the malignant tissues [47], [48]. In a study by Kagara et al., highly metastatic and advanced breast cancers showed higher expression of ZIP10 than non-metastatic breast cancers [49]. Furthermore, the study suggests that in advanced breast cancers, which express high levels of ZIP10, larger amounts of zinc are transported into the cell cytosol from the serum leading to metastasis. In the present study, we show that ZIP10 localizes to the plasma membrane of hepatocytes and zinc transport is inhibited by Zip10 siRNA. Additionally, preliminary data collected during this study suggests that knocking down Zip10 abundance inhibits E2F (a regulator of cell cycle progression) [50] activity (Figure S3), and decreases expression of the proliferative marker genes thymidine kinase (*tk*) and dihydrofolate reductase (*dhfr*) [51] (Lichten et al., unpublished observations). Taken together, these findings may reveal a role for Zip10 in not only cancer metastasis, but in cell cycle progression, and proliferation.

Our finding of the high Zip10 expression in mouse brain suggests that ZIP10 transport may be critical to maintaining neuronal functions and that the regional expression has teleologic relevance. Zinc is believed to have second messenger functions in brain and the ZIP transporters are believed to be key to cellular homeostasis [16], [52]. Of relevance is the suggestion of the evolutionary link of ZIP5, 6, and 10 ectodomains with the prion proteins [53]. This phylogenetic connection could lead to an understanding of ZIP10 as a factor in neurologic disease.

In summary, the experiments described here using intact mice and isolated murine hepatocytes show that MTF-1 is an integral part of ZIP10-related cellular zinc homeostasis in the liver both during zinc restriction and zinc excess. The results show that MTF-1 has physiologic significance and can act as a repressor of *Zip10* under normal cellular levels of labile zinc. Upon reducing cellular zinc, repression is lost, as MTF-1 is not translocated to the nucleus allowing enhanced *Zip10* transcriptional activation. The apparent differential mode of MTF-1 action, resides in the genomic placement of the MRE downstream of the *Zip10* transcription start site. The results also show that ZIP10 is a new target to investigate dietary influences on zinc metabolism by the liver and within specific regions of the brain and therein could help to elucidate the neurobiological effects of zinc that have been elusive thus far.

## Supporting Information

Figure S1 **The relative difference in Zip10 expression between liver and brain tissues.** The transcript abundance of Zip10 from each tissue was analyzed by qRT-PCR. Zip10 expression was normalized to 18s rRNA. n = 5.
(TIF)

Figure S2 **5′ RACE analysis of the** ***Zip10*** **transcription start site.** Total RNA was collected from liver tissue and reverse transcribed to cDNA using transcript specific primers for Zip10 mRNA. The cDNA was then cloned and sequenced. (**A**) The sequence obtained from the public record matches the sequence obtained by 5′RACE. (B) Agarose gel analysis of 5′ RACE product.
(TIF)

Figure S3 **Zip10 siRNA affects signalling pathways related to proliferation.** The Cignal 45 reporter array (QIAGEN) was used to identify signalling pathways that may be activated or repressed by knocking down Zip10 abundance. The top five activated and repressed pathways are shown.
(TIF)

Table S1 Average Ct values for *Zip* transporter genes.
(DOC)
