# Supplementary figures and images for: MTF-1-Mediated Repression of the Zinc Transporter Zip10 Is Alleviated by Zinc Restriction
Source: PLoS One. 2011 Jun 27;6(6):e21526. doi: 10.1371/journal.pone.0021526 (PMC3124522; doi:10.1371/journal.pone.0021526)

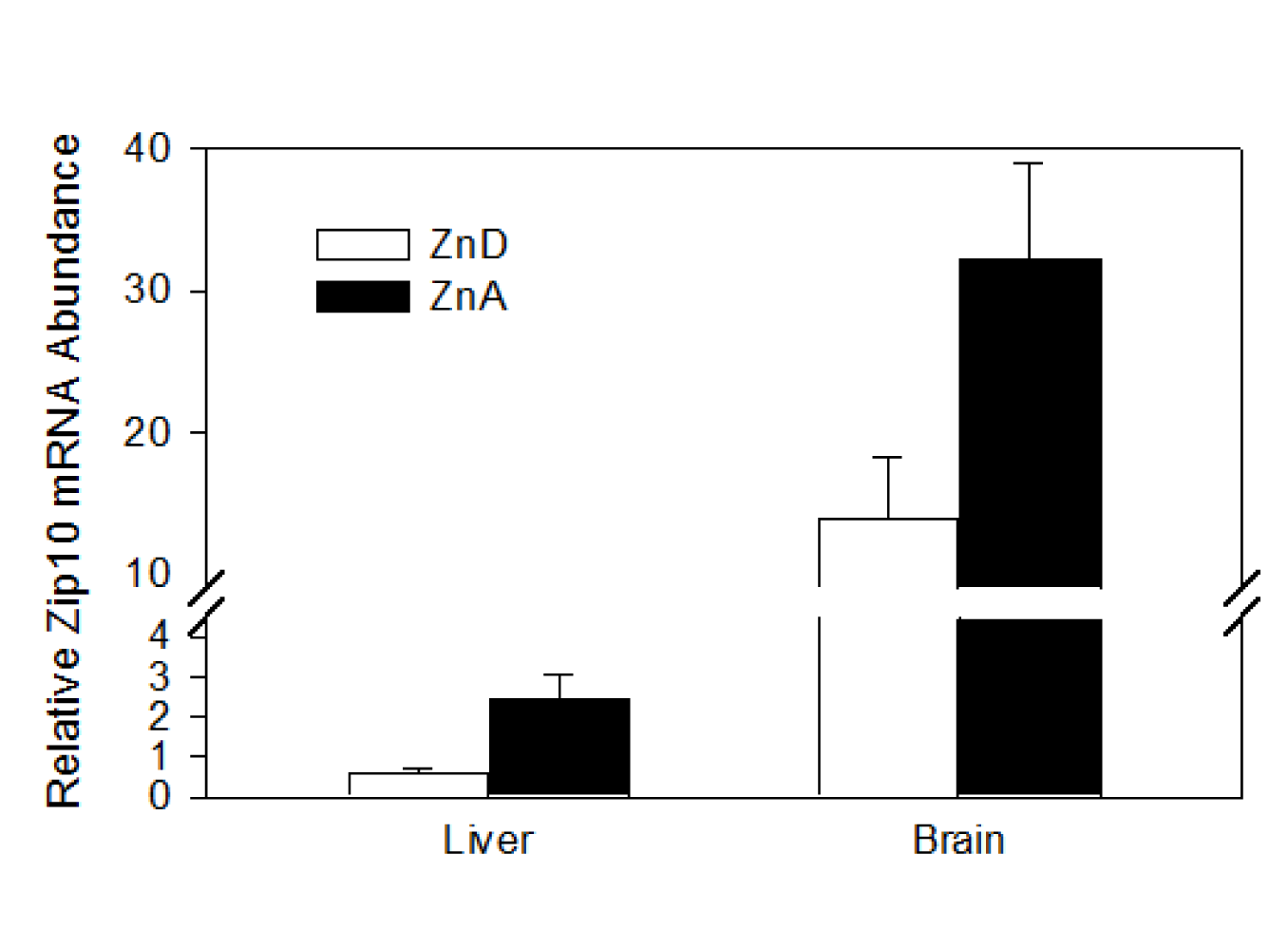

Supplement: Figure S1 — The relative difference in Zip10 expression between liver and brain tissues. The transcript abundance of Zip10 from each tissue was analyzed by qRT-PCR. Zip10 expression was normalized to 18s rRNA. n = 5. (TIF) [file pone.0021526.s001.tif]

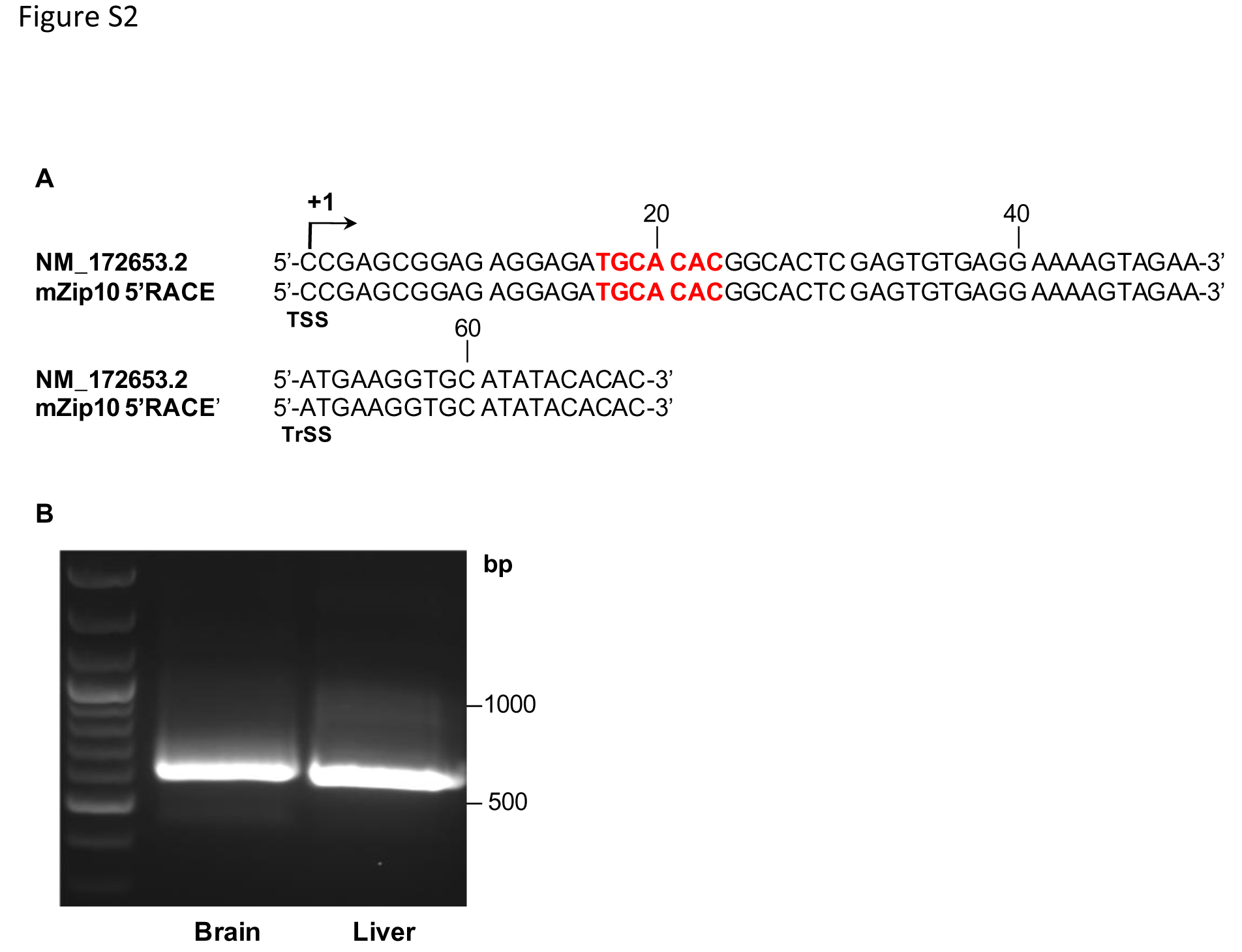

Supplement: Figure S2 — 5′ RACE analysis of the Zip10 transcription start site. Total RNA was collected from liver tissue and reverse transcribed to cDNA using transcript specific primers for Zip10 mRNA. The cDNA was then cloned and sequenced. (A) The sequence obtained from the public record matches the sequence obtained by 5′RACE. (B) Agarose gel analysis of 5′ RACE product. (TIF) [file pone.0021526.s002.tif]

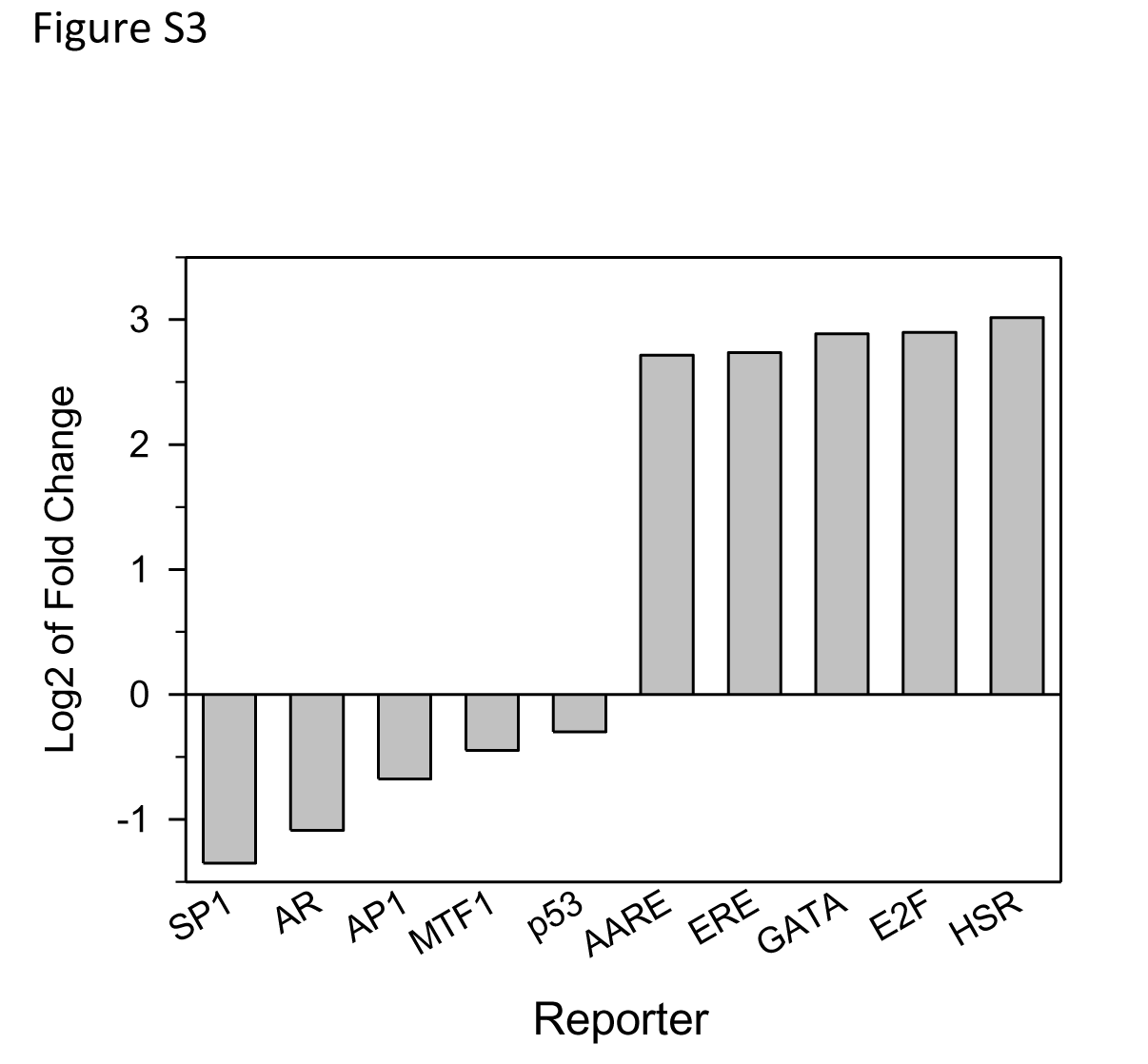

Supplement: Figure S3 — Zip10 siRNA affects signalling pathways related to proliferation. The Cignal 45 reporter array (QIAGEN) was used to identify signalling pathways that may be activated or repressed by knocking down Zip10 abundance. The top five activated and repressed pathways are shown. (TIF) [file pone.0021526.s003.tif]
