# Supplementary material for: MTF-1-Mediated Repression of the Zinc Transporter Zip10 Is Alleviated by Zinc Restriction
Source: PLoS One. 2011 Jun 27;6(6):e21526. doi: 10.1371/journal.pone.0021526 (PMC3124522; doi:10.1371/journal.pone.0021526)
Supplement: Table S1 — Average Ct values for Zip transporter genes. (DOC) [file pone.0021526.s004.doc]

**Table S1. Average Ct values for *Zip* transporter genes.**

Each Zip assay included: a standard curve of 5 serial dilution points of total RNA (ranging from 25 ng to 40 pg), a no-template control, and 5 ng of each sample RNA. Average Ct values for 5 ng of liver and brain are shown. The limit of detection (LOD) (Ct value) for each individual assay is shown for reference. All PCR efficiencies were between 90% and 110%.

| **Assay** | **Liver Ct Avg** | **Brain Ct Avg** | **LOD** |
| --- | --- | --- | --- |
|  | | | |
| Zip1 | 30 | 30 | 35.8 |
| Zip2 | 31 | 30 | 31.5 |
| Zip3 | 28 | 25 | 35.4 |
| Zip4 | 27 | 30 | 32.9 |
| Zip5 | 31 | 30 | 36 |
| Zip6 | 29 | 24 | 38 |
| Zip7 | 23 | 23 | 33 |
| Zip8 | 26 | 28 | 32 |
| Zip9 | 28 | 30 | 34 |
| Zip10 | 29 | 24 | 34 |
| Zip11 | 27 | 28 | 34.4 |
| Zip12 | 32 | 33 | 36 |
| Zip13 | 27 | 29 | 33 |
| Zip14 | 24 | 27 | 36 |
